# Adipose-Derived Stem Cells Inhibited the Proliferation of Bladder Tumor Cells by S Phase Arrest and Wnt/β-Catenin Pathway

**DOI:** 10.1089/cell.2019.0047

**Published:** 2019-12-04

**Authors:** Tao Wang, Xi Yu, Jian Lin, Cong Qin, Tao Bai, Tao Xu, Lei Wang, Xiuheng Liu, Shenglan Li

**Affiliations:** ^1^ Department of Urology, Renmin Hospital of Wuhan University, Wuhan, China.; ^2^ Department of Urology, Peking University First Hospital, Beijing, China.; ^3^ Department of Radiography, Renmin Hospital of Wuhan University, Wuhan, China.

**Keywords:** adipose-derived stem cell, bladder tumor, S phase arrest, caspase3/7, Wnt/β-catenin

## Abstract

Adipose-derived stem cells (ADSCs), which are present in most organs and tissues, were evaluated as a novel medium for stem cell therapy. In this study, we investigated the effects and underlying mechanisms of ADSCs in bladder tumor (BT) cells. SV-HUC, T24, and EJ cells were cultured with ADSCs and conditioned medium from ADSCs (ADSC-CM). We observed that in routine culture, ADSCs significantly inhibited the proliferation of T24 and EJ cells in a dose-dependent manner. In addition, ADSC-CM attenuated the viability of T24 and EJ cells in a dose-dependent manner. Cell cycle analysis indicated that ADSC-CM was capable of inducing T24 and EJ cells S phase arrest and downregulating the expression of CDK 1, whereas the expression of cyclin A was increased. ADSC-CM could induce apoptosis in T24 cells. The mechanism of this effect likely involved the caspase3/7 pathway and Wnt/β-catenin pathway. These findings demonstrated that ADSCs could inhibit the proliferation of BT cells via secretory factors.

## Introduction

In 2018, bladder tumor (BT) was the second most common malignancy of the genitourinary tract with almost 80,000 newly diagnosed cases and almost 17,000 deaths in the USA (Siegel et al., 2019). The treatment schemes for BT such as surgical techniques, including minimally invasive surgery are various, and there has been an improvement in the understanding of multimodal treatments involving radiotherapy, chemotherapy, and immunotherapy (DeGeorge et al., 2017). These treatments are considered costly and bring a huge economic burden to patients. The high recurrence rate and unsatisfactory 5-year overall survival rate for BT are still in need of improvement (Jin et al., 2014). Thus, novel treatments for BT need to be identified.

Adipose-derived stem cells (ADSCs) isolated from the stromal vascular fraction of adipose tissue share the same characteristics as mesenchymal stem cells (MSCs) and can differentiate into adipogenic, myogenic, osteogenic, chondrogenic, and neurogenic cells (Nielsen et al., 2018; Zachar et al., 2011). Currently, many studies have linked many important growth factors, cytokines, and chemokines secreted by ADSCs and MSCs to cancer development and progression (Chen et al., 2018; Chu et al., 2018; Gazdic et al., 2017; Lu et al., 2016). However, there is debate over the ability of ADSCs to support or suppress tumor cell proliferation (Chu et al., 2015; Jing et al., 2016; Yu et al., 2015).

The present study was designed to reveal the effects of ADSCs on the growth of BT cells and to explore the underlying mechanisms of these effects. In this study, we provide evidence that secretome of ADSCs were able to influence T24 cells and EJ cells proliferation/apoptosis by S phase arrest and Wnt/β-catenin pathway. Maybe ADSCs secretome can be used as a treatment for BT patients in the future.

## Materials and Methods

### Chemicals and reagents

Collagenase I was purchased from Sigma-Aldrich (St. Quentin Fallavier, France). Fetal bovine serum (FBS) for ADSCs was purchased from Gibco (Paris, France). Trypsin, Dulbecco's modified Eagle's medium with high glucose (DMEM), penicillin, streptomycin, and phosphate-buffered saline (PBS) were provided by HyClone (Cergy-Pontoise, France).

### ADSC preparation and culture

Adipose tissue was obtained from the subcutaneous fat of 10 patients who underwent renal resection without tumor at the Department of Urology in Renmin Hospital of Wuhan University. All donors provided written informed consent. This study was conducted according to institutional guidelines and an approved protocol. ADSCs were isolated and cultured as we previously described (Yu et al., 2015). The researcher washed adipose tissue samples with sterile PBS to remove debris and red blood cells and then cut them into tiny pieces.

The tissues were digested by 0.1% collagenase I in DMEM at 37°C for 60 minutes with gentle agitation. Then, the mixture was centrifuged for 10 minutes at 1000 r/min. The cellular precipitate was then resuspended and filtered through a 100-m mesh filter. The filtrate was plated onto cell culture plates in the indicated culture medium (DMEM with 10% FBS) and maintained in an incubator at 37°C in 5% CO_2_ in the indicated culture medium (DMEM with 10% FBS). Cells at passages 3–6 were used for subsequent experiments.

### Cancer cell culture

SV-HUC, T24, and EJ cells were purchased from the Institute of Urology, Peking University. SV-HUC cells were cultured in F-12K containing 10% FBS. T24 and EJ cells were cultured in DMEM containing 10% FBS. Cells were grown in an incubator at 37°C in 5% CO_2_.

### Conditioned medium collection

About 5 × 10^4^ cells were cultured in complete medium in 6-well plates for 24 hours. The conditioned medium was collected from cultured cells after incubation in a serum-free medium for 24 hours. The medium was then filtered and used immediately, or stored at −80°C.

### Cell number assessment

Cells were cultured in 6-well plates for 24 hours, before serum starvation for 16 hours. Test groups were cocultured with ADSCs in a 1.0 μm transwell membrane for another 48 hours. Control groups were cultured with tumor cells. Cell number was counted by using a cell counter model (Muse).

### Cell viability assay

Cells were cultured in 96-well plates for 24 hours, before serum starvation for 16 hours. Test groups were cultured in ADSC-CM for another 48 hours in triplicate. Control groups were cultured in normal medium. Cell viability was then measured by MTS assay (CellTiter96 AQueous Assay; Promega, France).

### Cell cycle distribution analysis

Cells were cultured at concentrations to yield 40%–50% before treated with ADSC-CM or normal medium. After 48 hours, cells were harvested and washed twice with PBS, then centrifuged. The samples were fixed with 70% ethanol for at least 12 hours at 4°C. The cells were washed with PBS once to remove the ethanol and resuspended with propidium iodide solution (0.05 mg/mL) containing Triton-100 and RNase, incubated at 37°C for 30 minutes in the dark. DNA content was then analyzed using the FC500 flow cytometer.

### Western blot analysis

Cells were cultured for 48 hours with ADSC-CM or normal medium. Total protein extracts were obtained by lysing cells in cold radioimmunoprecipitation assay (RIPA) buffer with 1% phenylmethanesulfonyl fluoride (PMSF). Equal amounts of cell extract were resolved by SDS-PAGE and transferred onto nitrocellulose filter membrane (Millipore, Bedford, MA) and blocked with 5% nonfat milk in Tris-buffered saline (TBS)/Tween (0.05% Tween-20 in TBS). Blots were probed with glyceraldehyde-3-phosphate dehydrogenase (GAPDH), Cyclin A, CDK1, and β-catenin antibodies (CST, Beverly, MA). At last, they were developed using a Chemiluminescence Kit (Millipore) with G:BOX Chemi XL1. GENESys (Syngene, Cambridge, UK).

### Apoptosis assay

Cells were harvested after been treated with the ADSC-CM or T24-CM for 48 hours, washed with PBS twice, resuspended in 1 × binding buffer at a concentration of about 1 × 10^6^ cells/mL. We used a FITC Annexin V Apoptosis Detection Kit I (BD Biosciences). Five micro liter annexin V–FITC and 5 μL 7-AAD was added into 100 μL solution and left it in the dark for 15 minutes. Then 400 μL 1 × binding buffer was added into the mixture. Cells were analyzed by flow cytometry using CELL Quest Pro software.

### Assessment of activated caspase-3/7

A caspase-3/7 activation assay was performed using a Caspase-Glo™ 3/7 Assay Kit (Promega, France) in accordance with the protocol. T24 cells were cultured in 24-well plates for 24 hours, before serum starvation for 16 hours. Test groups were cultured in ADSC-CM for another 48 hours, while control groups were cultured in T24-CM. Caspase-Glo 3/7 reagent (100 μL) was then added to each well, and the plate was incubated at room temperature for 1 hour. The luminescence of each sample was measured with a GloMax-Multi Detection System (Promega).

### Statistical analysis

All data were analyzed by SPSS software (version 20.0). The results are expressed as the mean ± standard deviation. Statistical analyses were performed using Student's *t*-test. *p* < 0.05 was considered significant. All means were calculated from at least three independent experiments.

## Results

### ADSCs inhibited the proliferation and viability of T24 and EJ cells

**FIG. 1.**
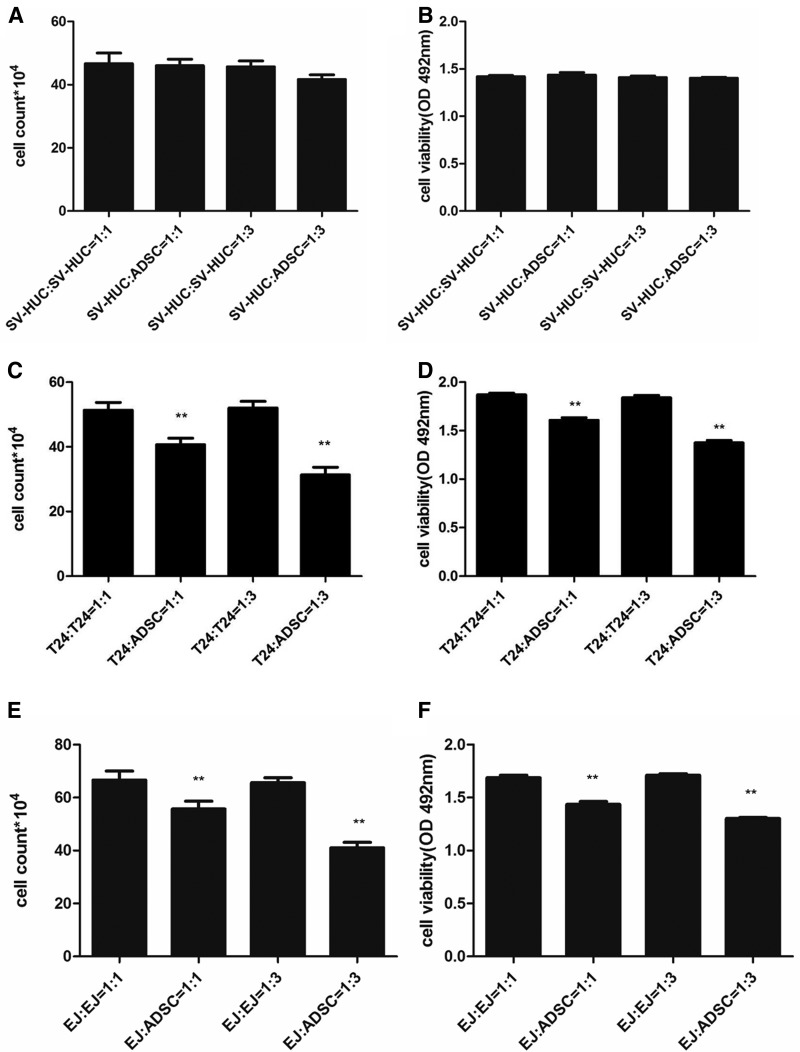
Effect of ADSCs on the growth and viability of SV-HUC, T24, and EJ cells. SV-HUC, T24, and EJ cells were, respectively, cocultured with SV-HUC, T24, and EJ or ADSCs at ratios of 1:1 and 3:1. The growth **(A)** or viability **(B)** of SV-HUC treated by ADSCs or ADSC-CM showed no significance versus control. The number of T24 **(C)** and EJ **(E)** cells was less in the treated group than the control group in dose-dependent manner. T24 **(D)** and EJ **(F)** cells viability was also inhibited by ADSC-CM in dose-dependent manner. Data shown are the mean ± SD of three independent experiments (**p < 0.01 vs. control). ADSCs, adipose-derived stem cells; SD, standard deviation.

ADSCs had a significant inhibitory effect on the proliferation of T24 and EJ cells. The number was not reduced when SV-HUC cells were cocultured with ADSCs (Fig. 1A), while a significant dose-dependent reduction in T24 (Fig. 1C) and EJ (Fig. 1E) cells number was observed at both 1:3 and 1:1 ratios with ADSCs. Next, we examined the viability of cells in the presence of ADSC-CM. As shown, ADSC-CM showed no significant inhibition in SV-HUC (Fig. 1B) cells, but inhibited the viability of T24 (Fig. 1D) and EJ (Fig. 1F) cells in a dose-dependent manner compared to that observed in control cells.

### ADSC-CM interfered with T24 and EJ cells proliferation by inducing cell cycle arrest

**FIG. 2.**
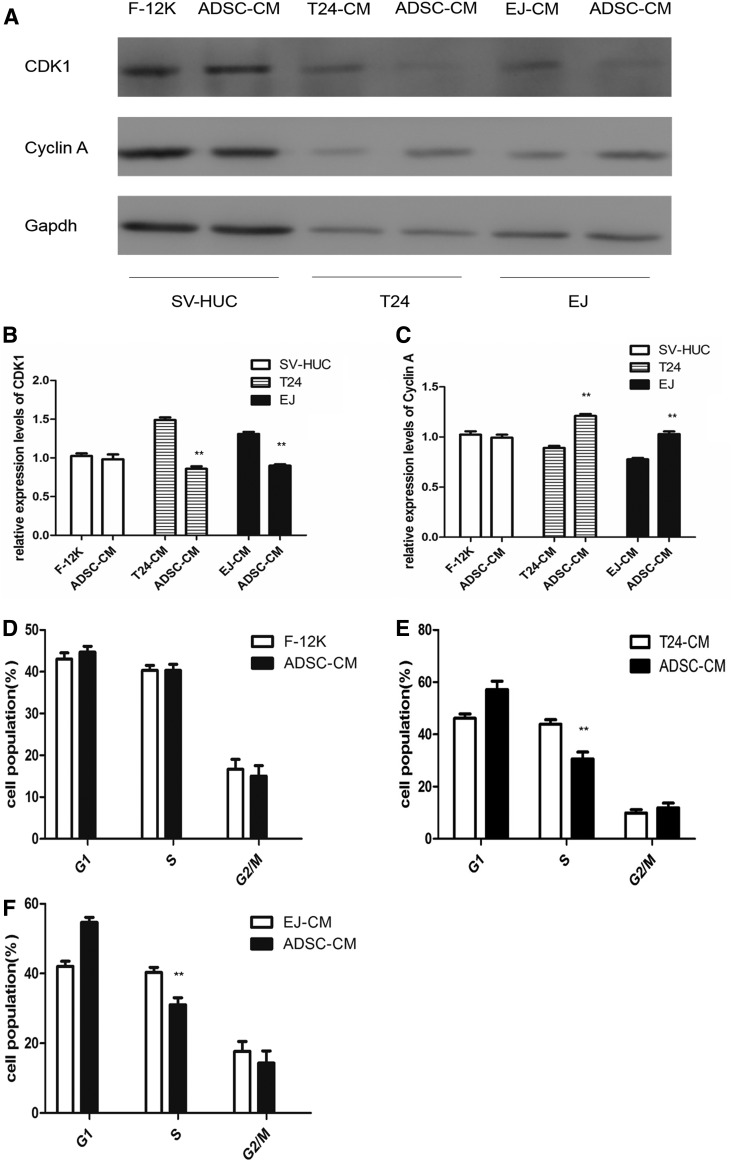
The western blot showed that cyclin A level was increased and CDK1 level was decreased in ADSC-CM-treated T24 and EJ cells, but not in SV-HUC cells **(A)**. Densitometry analysis of western blots showed quantitation of CDK1 **(B)** and cyclin A **(C)** levels. Cell cycle analysis of SV-HUC, T24, and EJ cells. There is no significance in SV-HUC groups **(D)**. It shows an obvious S phase arrest with a concomitant decrease of cell number in G1 phase of the cell cycle in ADSC-CM-treated T24 **(E)** and EJ **(F)** cells. (**p < 0.01 vs. control).

Western blotting was carried out to detect the impact of ADSC-CM on the expression of cyclin A and CDK1. The western blot showed that cyclin A level was increased and CDK1 level was decreased in ADSC-CM-treated T24 and EJ cells, but not in SV-HUC cells (Fig. 2A).

The expression of CDK1 (Fig. 2B) was decreased, whereas cyclin A (Fig. 2C) was increased in the ADSC-CM groups, suggesting that ADSC-CM evoked S phase arrest in T24 and EJ cells possibly through the dysregulation of CDK1 and cyclin A. Cell cycle analysis showed that there is no significance in SV-HUC groups (Fig. 2D), while 43.8% of the T 24 cells in the ADSC-CM groups were arrested at the S phase, 7.7% were arrested at the G2/M phase, and 48.5% were arrested at the G1 phase of the cell cycle, whereas 30.8% of the cells in T24-CM groups were arrested at the S phase, 8.7% were arrested at the G2/M phase, and 61.5% were arrested at the G1 phase of the cell cycle (Fig. 2E). The similar result was carried out in T24-CM-treated EJ cells (Fig. 2F).

### Induction of apoptosis in T24 cells by ADSC-CM

**FIG. 3.**
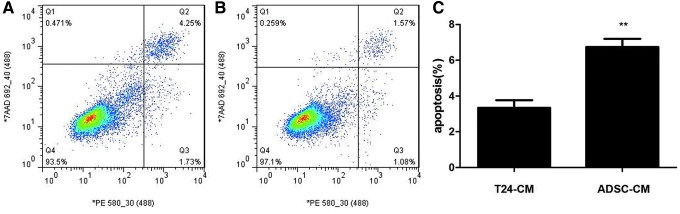
ADSC-CM treatment induces apoptosis in T24 cells. Cells were analyzed by flow cytometry. The gate setting distinguished between living (*bottom left*), necrotic (*top left*), early apoptotic (*bottom right*), and late apoptotic (*top right*) cells. The apoptosis of T24 cells was increased in the ADSC-CM groups **(A)** than the T24-CM groups **(B)** from three independent experiments **(C)**. (**p < 0.01 vs. control).

Despite the inhibitory effects of ADSC-CM on proliferation and viability mentioned above as well as the ability of ADSC-CM to induce S phase arrest, we speculated whether ADSC-CM also induced apoptosis in T24 cells. Apoptosis was observed more frequently in the ADSC-CM groups than in T24-CM groups. The ratio of apoptotic cells was significantly higher in the ADSC-CM groups (Fig. 3A) than in the T24-CM groups (Fig. 3B). Consistent with the results of MTS assay, these results revealed that ADSC-CM induced cellular apoptosis (Fig. 3C).

The mechanism of apoptosis in T24 cells induced by ADSC-CM. Caspase 3/7 was activated when cells underwent apoptosis.

**FIG. 4.**
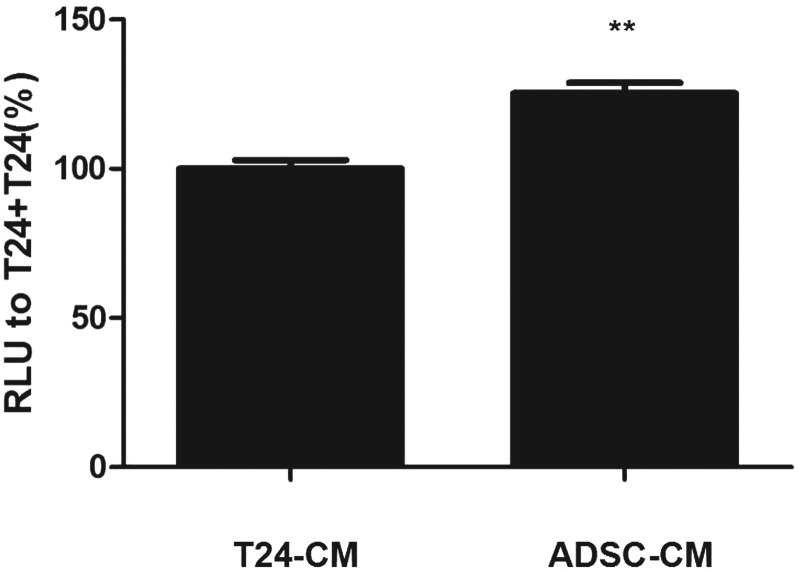
Caspase 3/7 was measured in T24 cells treated with ADSC-CM or T24-CM. RLU of the cells with ADSC-CM to those with T24-CM was expressed as caspase 3/7 activity. (**p < 0.01 vs. control). RLU, relative luminescence.

In the ADSC-CM groups, caspase 3/7 expression was significantly increased compared with that in the T24-CM groups (Fig. 4).

### ADSC-CM activates Wnt/β-catenin signaling in T24 cells

**FIG. 5.**
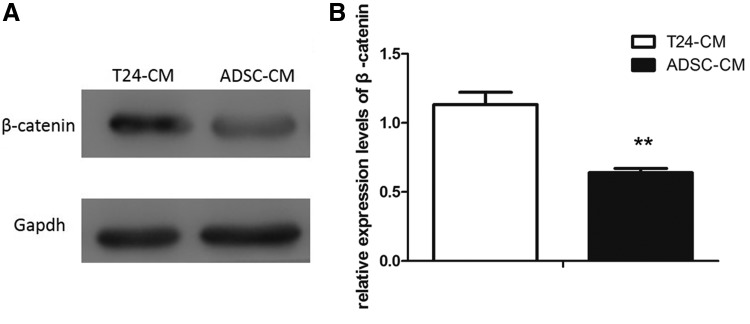
The western blot showed β-catenin level was decreased in ADSC-CM groups **(A)**. Densitometry analysis of western blots showed quantitation of β-catenin level **(B)**. (**p < 0.01 vs. control).

To determine the association between ADSC-CM and the activation level of Wnt/β-catenin signaling pathway, western blot analysis was used to assess the impacts of ADSC-CM on Wnt/β-catenin signaling pathway in T24 cells. The expression of β-catenin was shown. While cultured in ADSC-CM, the expression of β-catenin was less than being cultured in T24-CM (Fig. 5A, B). The result indicated that ADSC-CM could affect the activity of Wnt/β-catenin pathway in T24 cells.

## Discussion

In a series of experiments, we examined the antiproliferative and proapoptotic effects of ADSCs on T24 and EJ cells. Coculture experiments suggested that ADSCs directly inhibited T24 and EJ cell proliferation in a dose-dependent manner. Then, we treated T24 and EJ cells with ADSC-CM to determine whether some secreted factors were involved in this inhibition. We found that ADSC-CM attenuated T24 and EJ cell viability in a dose-dependent manner.

These results suggested that ADSCs may secrete some soluble factors that influence T24 and EJ cell growth. Many studies have reported that the soluble factors of ADSCs (such as DKK-1, SDF-1, TGF-β. and RANTES), prostaglandins (such as PGE2, PGI2 and PGJ2) or interleukins (such as IL6, IL8, GSF, and IL11) have different effects on different tumors (Lamfers et al., 2009; Vilalta et al., 2009; Yu et al., 2015; Zhu et al., 2009).

Hariharan found that IL6 secreted by adipose tissue influenced the migration of BT cells and enhanced tumor progression (Hariharan et al., 2018). The researches on ADSC originated IL6 showed that it was a key promoter of BT progression (Chu et al., 2018; Lu et al., 2016). DKK-1, SDF-1, TGF-β, and RANTES have been found to increase the risk of developing bladder cancer in lots of researches (Fan et al., 2014; Kumari et al., 2017; Sun et al., 2015; Zhang et al., 2018).

However, this research based on the use of a conditioned medium suggested that the paracrine activity of ADSCs exert antitumor progression effects. We did not know which soluble factor was responsible for the observed effects. May be it referred to extracellular vesicles (EVs), such as apoptotic bodies and exosomes. Wu reported that EVs from human umbilical cord Wharton's jelly MSCs reversed the development of BT cells possibly by downregulating phosphorylation of Akt and upregulating cleaved caspase-3 (Wu et al., 2013).

Another research showed that ADSCs-derived exosomes inhibited prostate cancer via delivery of miR-145 by promoting apoptosis through the caspase-3/7 pathway and reducing the activity of Bcl-xL (Takahara et al., 2016). In the same way, EVs were found to inhibit proliferation and to promote apoptosis in Kaposi's sarcoma, liver carcinoma, and ovarian tumor cell lines (Bruno et al., 2013). Higher miRNA content as well as superior amounts of cytokines and adhesion molecules in exosomes might also be involved in the tumor-promoting effects. So, we intended to isolate exosome for further research.

According to the above results, ADSCs had an inhibitory impact on T24 and EJ cell proliferation. To discover the mechanism involved, we conducted cell cycle analysis using flow cytometry. Cell cycle analyses demonstrated that ADSC-CM exposure caused S-phase arrest in T24 and EJ cells and deregulation of cyclin A and CDK1, which are essential for the S to G2/M transition. ADSC-CM-treated T24 and EJ cells did not progress into G2-M phase, were arrested in S phase, failed to accomplish normal mitosis, and ultimately perished by apoptosis.

Cyclin A accumulated at the G1/S phase transition and persisted through S phase. Cyclin A was initially associated with CDK2 in the late S phase, and was then associated with CDK1. Cyclin A-associated kinase activity is required for entry into S phase, the completion of S phase, and entry into M phase (Wenzel and Singh, 2018). Cyclin A and CDK1 dysfunction provides an additional explanation for the S phase arrest induced by ADSC-CM. There was also some evidences that cyclin A and CDK1 began to form in late S phase and were overexpressed in G2 phase, resulting in the progression from S phase to G2/M phase (Braun and Zischka, 2008; Yam et al., 2002; Zheng and Yu, 2018).

Apoptosis and cell cycle arrest are two different mechanisms involved in the induction of cell death (Choi et al., 2018; Jung et al., 2019; Masuda et al., 2018; Salha et al., 2018; Zhang et al., 2010). We found increased apoptosis in T24 cells in the ADSC-CM groups than those in the T24-CM groups and that caspase 3/7 expression was significantly increased. Among the caspase family, caspase 1, caspase 11, and possibly caspase 4, are not directly involved in the transduction of apoptotic signals, they are mainly involved in the activation of interleukin precursors; caspase 2, caspase 8, caspase 9, and caspase 10 were involved. The initiation of apoptosis: caspase 3, caspase 6, and caspase 7 are involved in the initiation of apoptosis, and caspase 3 and 7 have similar substrate polymerases that are involved in DNA repair and monitor gene integrity.

At the initiation of apoptosis, the 116 kDa PARP protein is cut between Asp216-Gly217 into 31 kDa and 85 kDa fragments by caspase-3, making two zinc finger structures that bind to the DNA of PARP separated from the catalytic region at the carboxy terminus, which fails to function normally. As a result, the activity of Ca^2+^/Mg^2+^-dependent endonuclease, which is affected by the negative regulation of PARP, is increased, and DNA between nucleosomes is cleaved, causing apoptosis (Julien and Wells, 2017; Kim and Kim, 2018; Orning et al., 2018; Zeng et al., 2018; Zhu and Li, 2018). Based on these results, we suggest that ADSC-CM induced T24 cell apoptosis via the caspase-3/7 pathway.

Aberrant activation of the Wnt/β-catenin signaling pathway often occurred during the initiation and progression of BT (Chen et al., 2018b; Pei et al., 2017). As we know, Wnt/β-catenin pathway was inactive due to inhibition of β-catenin expression and translocation to nucleus. Wnt/β-catenin signaling is one of the important molecular mechanisms to regulate cell proliferation, invasion, and differentiation through β-catenin protein, which is an important signaling molecule in the Wnt/β-catenin pathway (Sumithra et al., 2016; Tai et al., 2015). β-catenin accumulates in the cytoplasm and translocates to the nucleus, and then activates downstream target genes, such as c-myc and cyclin D1. In our study, we observed that ADSC-CM inhibited the nuclear translocation of β-catenin in T24 cell, which leads to suppression of Wnt/β-catenin signaling pathway.

## Conclusion

In summary, our results demonstrated that ADSCs could obviously inhibit the proliferation of T24 and EJ cells in a dose-dependent manner through apoptosis and S-phase arrest. The antiproliferative effect of ADSCs appeared to be associated with the secretion of soluble factors that were involved with caspase-3/7 and Wnt/β-catenin signaling pathway. Further research was needed to clarify which soluble factor or EVs worked and whether it possessed the same effect on other BT *in vivo*.

## References

[B1] Braun R.J., and Zischka H. (2008). Mechanisms of Cdc48/VCP-mediated cell death: from yeast apoptosis to human disease. Biochim. Biophys. Acta 1783, 1418–1435 18284922 10.1016/j.bbamcr.2008.01.015

[B2] Bruno S., Collino F., Deregibus M.C., Grange C., Tetta C., and Camussi G. (2013). Microvesicles derived from human bone marrow mesenchymal stem cells inhibit tumor growth. Stem Cells Dev. 22, 758–771 23034046 10.1089/scd.2012.0304

[B3] Chen B., Yu J., Wang Q., Zhao Y., Sun L., Xu C., Zhao X., Shen B., Wang M., Xu W., and Zhu W. (2018a). Human bone marrow mesenchymal stem cells promote gastric cancer growth via regulating c-Myc. Stem Cells Int. 2018, 9501747 10.1155/2018/9501747 PMC6116400 30186330

[B4] Chen Z., Zhou L., Wang L., Kazobinka G., Zhang X., Han X., Li B., and Hou T. (2018b). HBO1 promotes cell proliferation in bladder cancer via activation of Wnt/beta-catenin signaling. Mol. Carcinog. 57, 12–21 28796367 10.1002/mc.22715

[B5] Choi S.A., Lee C., Kwak P.A., Park C.K., Wang K.C., Phi J.H., Lee J.Y., Chong S., and Kim S.K. (2018). Histone deacetylase inhibitor panobinostat potentiates the anti-cancer effects of mesenchymal stem cell-based sTRAIL gene therapy against malignant glioma. Cancer Lett. 442, 161–169 30367915 10.1016/j.canlet.2018.10.012

[B6] Chu Y., Tang H., Guo Y., Guo J., Huang B., Fang F., Cai J., and Wang Z. (2015). Adipose-derived mesenchymal stem cells promote cell proliferation and invasion of epithelial ovarian cancer. Exp. Cell. Res. 337, 16–27 26209607 10.1016/j.yexcr.2015.07.020

[B7] Chu Y., Wang Y., Peng W., Xu L., Liu M., Li J., Hu X., Li Y., Zuo J., and Ye Y. (2018). STAT3 activation by IL-6 from adipose-derived stem cells promotes endometrial carcinoma proliferation and metastasis. Biochem. Biophys. Res. Commun. 500, 626–631 29684351 10.1016/j.bbrc.2018.04.121

[B8] DeGeorge K.C., Holt H.R., and Hodges S.C. (2017). Bladder cancer: diagnosis and treatment. Am. Fam. Physician. 96, 507–514 29094888

[B9] Fan Y., Shen B., Tan M., Mu X., Qin Y., Zhang F., and Liu Y. (2014). TGF-beta-induced upregulation of malat1 promotes bladder cancer metastasis by associating with suz12. Clin. Cancer. Res. 20, 1531–1541 24449823 10.1158/1078-0432.CCR-13-1455

[B10] Gazdic M., Simovic M.B., Jovicic N., Misirkic-Marjanovic M., Djonov V., Jakovljevic V., Arsenijevic N., Lukic M.L., and Volarevic V. (2017). Mesenchymal stem cells promote metastasis of lung cancer cells by downregulating systemic antitumor immune response. Stem Cells Int. 2017, 6294717 10.1155/2017/6294717 PMC5534320 28798777

[B11] Hariharan N., Ashcraft K.A., Svatek R.S., Livi C.B., Wilson D., Kaushik D., Leach R.J., and Johnson-Pais T.L. (2018). Adipose tissue-secreted factors alter bladder cancer cell migration. J. Obes. 2018, 9247864 10.1155/2018/9247864 PMC5985104 29887999

[B12] Jin X., Yun S.J., Jeong P., Kim I.Y., Kim W.J., and Park S. (2014). Diagnosis of bladder cancer and prediction of survival by urinary metabolomics. Oncotarget 5, 1635–1645 24721970 10.18632/oncotarget.1744 PMC4039236

[B13] Jing H.X., Duan D.J., Zhou H., Hu Q.M., and Lei T.C. (2016). Adiposederived mesenchymal stem cellfacilitated TRAIL expression in melanoma treatment in vitro. Mol. Med. Rep. 14, 195–201 27177242 10.3892/mmr.2016.5283 PMC4918625

[B14] Julien O., and Wells J.A. (2017). Caspases and their substrates. Cell Death Differ. 24, 1380–1389 28498362 10.1038/cdd.2017.44 PMC5520456

[B15] Jung P.Y., Ryu H., Rhee K.J., Hwang S., Lee C.G., Gwon S.Y., Kim J., Kim J., Yoo B.S., Baik S.K., Bae K.S., and Eom Y.W. (2019). Adipose tissue-derived mesenchymal stem cells cultured at high density express IFN-beta and TRAIL and suppress the growth of H460 human lung cancer cells. Cancer Lett. 440–441, 202–210 10.1016/j.canlet.2018.10.017 30393160

[B16] Kim H.M., and Kim Y.M. (2018). HMGB1: LPS delivery vehicle for caspase-11-mediated pyroptosis. Immunity 49, 582–584 30332623 10.1016/j.immuni.2018.09.021

[B17] Kumari N., Agrawal U., Mishra A.K., Kumar A., Vasudeva P., Mohanty N.K., and Saxena S. (2017). Predictive role of serum and urinary cytokines in invasion and recurrence of bladder cancer. Tumour Biol. 39, 1010428317697552 10.1177/1010428317697552 28378639

[B18] Lamfers M., Idema S., van Milligen F., Schouten T., van der Valk P., Vandertop P., Dirven C., and Noske D. (2009). Homing properties of adipose-derived stem cells to intracerebral glioma and the effects of adenovirus infection. Cancer Lett. 274, 78–87 18842332 10.1016/j.canlet.2008.08.035

[B19] Lu J.H., Wei H.J., Peng B.Y., Chou H.H., Chen W.H., Liu H.Y., and Deng W.P. (2016). Adipose-derived stem cells enhance cancer stem cell property and tumor formation capacity in Lewis lung carcinoma cells through an interleukin-6 paracrine circuit. Stem Cells Dev. 25, 1833–1842 27596042 10.1089/scd.2016.0163

[B20] Masuda J., Takayama E., Ichinohe T., Strober W., Mizuno-Kamiya M., Ikawa T., Kitani A., Kawaki H., Fuss I., Kawamoto H., Seno A., Vaidyanath A., Umemura N., Mizutani A., Kasai T., Honjo Y., Satoh A., Murakami H., Katsura Y., Kondoh N., and Seno M. (2018). Suppression effect on IFN-gamma of adipose tissue-derived mesenchymal stem cells isolated from beta2-microglobulin-deficient mice. Exp. Ther. Med. 16, 4277–4282 30344701 10.3892/etm.2018.6689 PMC6176164

[B21] Nielsen E.O., Chen L., Hansen J.O., Degn M., Overgaard S., and Ding M. (2018). Optimizing osteogenic differentiation of ovine adipose-derived stem cells by osteogenic induction medium and FGFb, BMP2, or NELL1 in vitro. Stem Cells Int. 2018, 9781393 10.1155/2018/9781393 PMC6178511 30356449

[B22] Orning P., Weng D., Starheim K., Ratner D., Best Z., Lee B., Brooks A., Xia S., Wu H., Kelliher M.A., Berger S.B., Gough P.J., Bertin J., Proulx M.M., Goguen J.D., Kayagaki N., Fitzgerald K.A., and Lien E. (2018). Pathogen blockade of TAK1 triggers caspase-8-dependent cleavage of gasdermin D and cell death. Science 362, 1064–1069 30361383 10.1126/science.aau2818 PMC6522129

[B23] Pei Z., Du X, Song Y., Fan L., Li F., Gao Y., Wu R., Chen Y., Li W., Zhou H., Yang Y., and Zeng J. (2017). Down-regulation of lncRNA CASC2 promotes cell proliferation and metastasis of bladder cancer by activation of the Wnt/beta-catenin signaling pathway. Oncotarget 8, 18145–18153 28199978 10.18632/oncotarget.15210 PMC5392314

[B24] Salha S., Gehmert S., Brebant V., Anker A., Loibl M., Prantl L., and Gehmert S. (2018). PDGF regulated migration of mesenchymal stem cells towards malignancy acts via the PI3K signaling pathway. Clin. Hemorheol. Microcirc. 70, 543–551 30347613 10.3233/CH-189319

[B25] Siegel R.L., Miller K.D., and Jemal A. (2019). Cancer statistics, 2019. CA Cancer J. Clin. 69, 7–34 30620402 10.3322/caac.21551

[B26] Sumithra B., Saxena U., and Das A.B. (2016). Alternative splicing within the Wnt signaling pathway: role in cancer development. Cell Oncol (Dordr). 39, 1–13 26762488 10.1007/s13402-015-0266-0 PMC13001886

[B27] Sun D.K., Wang L., Wang J.M., and Zhang P. (2015). Serum Dickkopf-1 levels as a clinical and prognostic factor in patients with bladder cancer. Genet. Mol. Res. 14, 18181–18187 26782465 10.4238/2015.December.23.5

[B28] Tai D., Wells K., Arcaroli J., Vanderbilt C., Aisner D.L., Messersmith W.A., and Lieu C.H. (2015). Targeting the WNT signaling pathway in cancer therapeutics. Oncologist 20, 1189–1198 26306903 10.1634/theoncologist.2015-0057 PMC4591954

[B29] Takahara K., Ii M., Inamoto T., Nakagawa T., Ibuki N., Yoshikawa Y., Tsujino T., Uchimoto T., Saito K., Takai T., Tanda N., Minami K., Uehara H., Komura K., Hirano H., Nomi H., Kiyama S., Asahi M., and Azuma H. (2016). microRNA-145 mediates the inhibitory effect of adipose tissue-derived stromal cells on prostate cancer. Stem Cells Dev. 25, 1290–1298 27465939 10.1089/scd.2016.0093

[B30] Vilalta M., Degano I.R., Bago J., Aguilar E., Gambhir S.S., Rubio N., and Blanco J. (2009). Human adipose tissue-derived mesenchymal stromal cells as vehicles for tumor bystander effect: a model based on bioluminescence imaging. Gene Ther. 16, 547–557 19092860 10.1038/gt.2008.176

[B31] Wenzel E.S., and Singh A. (2018). Cell-cycle checkpoints and aneuploidy on the path to cancer. In Vivo 32, 1–5 29275292 10.21873/invivo.11197 PMC5892633

[B32] Wu S., Ju G.Q., Du T, Zhu Y.J., and Liu G.H. (2013). Microvesicles derived from human umbilical cord Wharton's jelly mesenchymal stem cells attenuate bladder tumor cell growth in vitro and in vivo. PLoS One 8, e61366 10.1371/journal.pone.0061366 PMC3625149 23593475

[B33] Yam C.H., Fung T.K., and Poon R.Y. (2002). Cyclin A in cell cycle control and cancer. Cell. Mol. Life Sci. 59, 1317–1326 12363035 10.1007/s00018-002-8510-y PMC11337442

[B34] Yu X., Su B., Ge P., Wang Z., Li S., Huang B., Gong Y., and Lin J. (2015). Human adipose derived stem cells induced cell apoptosis and s phase arrest in bladder tumor. Stem Cells Int. 2015, 619290 10.1155/2015/619290 PMC4322296 25691904

[B35] Zachar V., Rasmussen J.G., and Fink T. (2011). Isolation and growth of adipose tissue-derived stem cells. Methods Mol. Biol. 698, 37–49 21431509 10.1007/978-1-60761-999-4_4

[B36] Zeng L., Qian J., Luo X., Zhou A., Zhang Z., and Fang Q. (2018). CHSY1 promoted proliferation and suppressed apoptosis in colorectal cancer through regulation of the NFkappaB and/or caspase-3/7 signaling pathway. Oncol. Lett. 16, 6140–6146 30344756 10.3892/ol.2018.9385 PMC6176353

[B37] Zhang T., Yang F., Li W., Liu B., Li W., Chen Z., and Wang C. (2018). Suppression of the SDF1/CXCR4/betacatenin axis contributes to bladder cancer cell growth inhibition in vitro and in vivo. Oncol. Rep. 40, 1666–1674 30015971 10.3892/or.2018.6546

[B38] Zhang Y., Bellows C.F., and Kolonin M.G. (2010). Adipose tissue-derived progenitor cells and cancer. World J. Stem Cells 2, 103–113 21607127 10.4252/wjsc.v2.i5.103 PMC3097931

[B39] Zheng G., and Yu H. (2018). Cyclin A turns on Bora to light the path to mitosis. Dev. Cell 45, 542–543 29870714 10.1016/j.devcel.2018.05.017

[B40] Zhu X., and Li S. (2018). TET2 inhibits tumorigenesis of breast cancer cells by regulating caspase-4. Sci. Rep. 8, 16167 10.1038/s41598-018-34462-z PMC6212556 30385776

[B41] Zhu Y., Sun Z., Han Q., Liao L., Wang J., Bian C., Li J., Yan X., Liu Y., Shao C., and Zhao R.C. (2009). Human mesenchymal stem cells inhibit cancer cell proliferation by secreting DKK-1. Leukemia 23, 925–933 19148141 10.1038/leu.2008.384

